# Sipuleucel-T immune parameters correlate with survival: an analysis of the randomized phase 3 clinical trials in men with castration-resistant prostate cancer

**DOI:** 10.1007/s00262-012-1317-2

**Published:** 2012-08-03

**Authors:** Nadeem A. Sheikh, Daniel Petrylak, Philip W. Kantoff, Corazon dela Rosa, Frances P. Stewart, Ling-Yu Kuan, James B. Whitmore, James B. Trager, Christian H. Poehlein, Mark W. Frohlich, David L. Urdal

**Affiliations:** 1 Dendreon Corporation, 1208 Eastlake Ave E, Seattle, WA 98102-3703 USA; 2 Department of Medicine, Columbia University, New York, NY 10032 USA; 3 Dana–Farber Cancer Institute, Harvard Medical School, Boston, MA 02215 USA

**Keywords:** Prostate cancer, Cellular, Humoral, Immunotherapy, Survival

## Abstract

**Purpose:**

Sipuleucel-T, the first FDA-approved autologous cellular immunotherapy for treatment of advanced prostate cancer, is manufactured by activating peripheral blood mononuclear cells, including antigen presenting cells (APCs), with a fusion protein containing prostatic acid phosphatase. Analysis of data from three phase 3 trials was performed to immunologically characterize this therapy during the course of the three doses, and to relate the immunological responses to overall survival (OS).

**Methods:**

Sipuleucel-T product characteristics [APC numbers, APC activation (CD54 upregulation), and total nucleated cell (TNC) numbers] were assessed in three randomized, controlled phase 3 studies (*N* = 737). Antigen-specific cellular and humoral responses were assessed in a subset of subjects. The relationships between these parameters and OS were assessed.

**Results:**

APC activation occurred in the first dose preparation [6.2-fold, (4.65, 7.70); median (25th, 75th percentile)] and increased in the second [10.6-fold (7.83, 13.65)] and third [10.5-fold (7.89, 13.65)] dose preparations. Cytokines and chemokines associated with activated APCs were produced during the manufacture of each dose; T-cell activation-associated cytokines were detected in the second and third dose preparations. Antigen-specific T cells were detectable after administration of the first sipuleucel-T dose. Cumulative APC activation, APC number, and TNC number correlated with OS (*P* < 0.05). Antigen-specific immune responses were observed in 78.8 % of monitored subjects and their presence correlated with OS (*P* = 0.003).

**Conclusion:**

Sipuleucel-T broadly engages the immune system by activating APCs ex vivo and inducing long-lived immune responses in vivo. These data indicate antigen-specific immune activation as a mechanism by which sipuleucel-T prolongs OS.

**Electronic supplementary material:**

The online version of this article (doi:10.1007/s00262-012-1317-2) contains supplementary material, which is available to authorized users.

## Introduction

Immunotherapy has long held the promise of controlling cancer; however, attempts to translate immunotherapeutic strategies into practice have been unsuccessful prior to the 2010 FDA approval of the autologous cellular immunotherapy, sipuleucel-T, in patients with asymptomatic or minimally symptomatic metastatic castration-resistant prostate cancer (mCRPC) [1–4]. Each dose of sipuleucel-T is manufactured by culturing an individual’s own freshly isolated peripheral blood mononuclear cells (PBMCs), including antigen presenting cells (APCs) and T cells, with a fusion protein (PA2024) composed of prostatic acid phosphatase (PAP; an antigen expressed in prostate adenocarcinoma [5, 6]) linked to granulocyte–macrophage colony-stimulating factor (GM-CSF; an APC activator [7, 8]). A course of therapy consists of three doses of sipuleucel-T administered at two-week intervals. Before infusion, each dose of sipuleucel-T is tested to ensure that it meets quality standards, including assessments of both cell viability and potency, by quantifying the number of APCs and the activation of these cells. Thus, the preparation of each sipuleucel-T dose provides an opportunity to immunologically characterize this autologous cellular immunotherapy as it develops over the course of the three doses.

Previous studies have shown low levels of basal immune response to PAP in prostate cancer patients [9–11], which were augmented following immunization [12, 13, 16]; however, the studies were not sufficient in size to explore correlations with overall survival (OS). In three phase 3, double-blind, placebo-controlled studies in subjects with mCRPC [14–16], treatment with sipuleucel-T prolonged OS, providing a unique opportunity to analyze how the product parameters that were assessed on each dose of sipuleucel-T, as well as peripheral immune responses that were monitored in a subset of these subjects, related to OS.

## Materials and methods

### Trial design and participants

The phase 3 trials IMPACT (*n* = 512) and D9901/D9902A (*n* = 225) were randomized, double-blind, multicenter studies of men with mCRPC randomized 2:1 to receive sipuleucel-T or control [14–16]. Sipuleucel-T product parameters were evaluated in all patients who received at least one infusion. In order to allow a detailed examination of the immune response to sipuleucel-T treatment, the IMPACT trial was amended to allow further characterization of the sipuleucel-T product in doses prepared at the primary manufacturing facility (Seattle, WA, USA), and to require peripheral immune response sampling at all clinical study sites. Thus, further detailed immunological characterization of the sipuleucel-T product was performed in a subset of consented IMPACT patients with available pre-culture cells (*N* = 20) and post-culture supernatants (*N* = 49) during the treatment phase. Peripheral immune responses were measured in a subset of consented subjects enrolled in the IMPACT study (*N* = 237). The studies were conducted in accordance with applicable regulations of the FDA and the Good Clinical Practice guidelines of the International Conference on Harmonisation and approved by the institutional review board at each study center.

### Treatment

Details of the manufacture of sipuleucel-T have been previously described [14–17]. In brief, sipuleucel-T was prepared by culturing freshly obtained leukapheresis PBMCs with PA2024 for 36–44 h at 37 °C. Control was prepared by culturing approximately one-third of PBMCs without PA2024; the remainder of the cells was cryopreserved for possible use following disease progression to manufacture a salvage treatment manufactured according to the same specifications as sipuleucel-T. Subjects received sipuleucel-T or control as an intravenous infusion over 30–60 min, approximately every 2 weeks for a total of three infusions.

### Product parameters

APC activation, APC number, and total nucleated cell (TNC) count were determined for every sipuleucel-T and control product as previously described [18], with APCs defined as large cells expressing CD54. APC activation was measured as the increase in surface CD54 on APCs, expressed as an upregulation ratio of the average number of molecules on post-culture versus pre-culture cells. Cumulative product parameters were defined as the sum of the values across the 3 treatment doses.

### Immunological characterization of the product

T-cell proliferation and interferon gamma (IFNγ) enzyme-linked immunosorbent spot (ELISPOT) were evaluated at treatment weeks 0, 2, and 4 for IMPACT subjects with remaining pre-culture cells (Supplementary Figure 1). Chemokines and cytokines were also measured in available post-culture supernatants of IMPACT subjects (Supplementary Figure 1).

#### Cellular proliferation

T-cell proliferation to PA2024 and PAP was assayed using a standard tritiated thymidine (^3^H-thymidine) incorporation assay [19]. The degree of proliferation was expressed as a stimulation index (SI), defined as ^3^H-thymidine incorporation in the presence of antigen divided by ^3^H-thymidine incorporation with media alone.

#### IFNγ ELISPOT

IFNγ ELISPOT assays were performed using polyvinylidene fluoride ELISPOT plates (Millipore, Billerica, MA, USA) and anti-human IFNγ antibodies (clone D1K and B6-1, MabTech, Upsala, Sweden) per the manufacturers’ instructions. ELISPOT data are presented as the median of triplicates with background (PBMCs incubated with media) IFNγ spots subtracted.

#### Ex vivo cytokine analysis

Ex vivo culture supernatant was analyzed for chemokine and cytokine content via Luminex assay (Biosource, Invitrogen Corp. Camarillo, CA, USA) or using a Mesoscale Discovery Sector Imager 2400 (Gaithersburg, MD, USA). In a subset of samples, pre-culture cells at each treatment week were cultured with recombinant GM-CSF (Leukine^®^ [sargramostim], Genzyme) and the culture medium was then assayed using the Mesoscale platform.

### Long-term peripheral immune responses

A subset of patients enrolled in IMPACT consented to provide blood for immune response determination at baseline (week 0) as well as 6, 14, and 26 weeks following the first infusion (100 mL maximum at each time point; Supplementary Figure 1); samples were processed within 24 h of collection. A 10-mL sample of blood was collected in coagulant-free tubes for serum isolation; the remainder was collected in sodium heparin-coated tubes to isolate PBMCs as described previously [17] and cryopreserved until batch assayed.

The T-cell proliferation and IFNγ ELISPOT assays were performed as described above. Sera were evaluated for the presence of antigen-specific antibodies via an initial enzyme-linked immunosorbent assay (ELISA) screening assay. ELISAs were performed for each serum sample in triplicate, and the geometric mean of the reciprocal of the dilution that yielded an optical density equivalent to assay background was reported.

Positive thresholds for treatment-related immune responses were selected in order to ensure that <5 % of subjects would exceed the value at baseline. Thresholds were: proliferation, SI > 12 for PA2024, >8 for PAP; IFNγ ELISPOT (per 3 × 10^5^ PBMC), >10 spots for PA2024, >40 spots for PAP; ELISA titer, >400 for both anti-PA2024 and anti-PAP antibodies. Serum samples that gave a positive response to an initial ELISA were subsequently evaluated for IgM and IgG antibody isotypes.

### Statistical analysis

T-cell proliferation and IFNγ ELISPOT were compared between groups using a *t*-test on the log-transformed median SI values and ranked median ELISPOT values. Antibody responses were compared between treatment groups using a Wilcoxon rank sum test of the antibody titers. Differences in T cell and antibody responder frequencies were compared between treatment groups using a Fisher’s exact test.

The correlation between OS and key cumulative product parameters was examined using a Cox regression model, with and without adjustment for baseline PSA and LDH [14–16, 20, 21]. Each of the parameters was log transformed, and the analysis was stratified by study. The correlation between OS and peripheral immune response responder status was examined using a Cox regression model, with and without adjustment for baseline PSA and LDH. All *P* values reported are two-tailed. No adjustment for multiplicity of endpoints or time points was made.

## Results

### Subjects and treatment

Of the 737 subjects randomized in the IMPACT, D9901, and D9902A studies, 476 received sipuleucel-T and 243 received control product, both with a median cell viability of >95 % across all 3 infusions. For the subset of subjects from IMPACT who provided blood for peripheral immune response determinations (*n* = 160 sipuleucel-T; *n* = 77 control), demographics and baseline disease characteristics were balanced between study arms and between the evaluated subgroup and the overall study population (Table 1).

**Table 1:** Subject demographics and baseline disease characteristics

	Sipuleucel-T		Control		Total	
	D9901 D9902A IMPACT (*N* = 488)	IMPACT Immune response subset (*N* = 160^a^)	D9901 D9902A IMPACT (*N* = 249)	IMPACT Immune response subset (*N* = 77^a^)	D9901 D9902A IMPACT (*N* = 737)	IMPACT Immune response subset (*N* = 237^a^)
Age (years)						
Median	72	72	71	70	71	71
Min, Max	47, 91	49, 89	40, 89	40, 87	40, 91	40, 89
Race *n* (%)						
Caucasian	437 (89.5 %)	138 (86.3 %)	229 (92.0 %)	68 (88.3 %)	666 (90.4 %)	206 (86.9 %)
Black or African American	33 (6.8 %)	13 (8.1 %)	10 (4.0 %)	5 (6.5 %)	43 (5.8 %)	18 (7.6 %)
Other	18 (3.7 %)	9 (5.6 %)	9 (3.6 %)	4 (5.2 %)	27 (3.7 %)	13 (5.5 %)
ECOG performance status *n* (%)						
0	393 (80.5 %)	126 (78.8 %)	199 (79.9 %)	65 (84.4 %)	592 (80.3 %)	191 (80.6 %)
1	95 (19.5 %)	34 (21.3 %)	50 (20.1 %)	12 (15.6 %)	145 (19.7 %)	46 (19.4 %)
Gleason sum *n* (%)						
≤6	74 (15.2 %)	16 (10.0 %)	31 (12.4 %)	8 (10.4 %)	105 (14.2 %)	24 (10.1 %)
≥7	413 (84.6 %)	144 (90.0 %)	217 (87.1 %)	69 (89.6 %)	630 (85.5 %)	213 (89.9 %)
>10 Bone metastases *n* (%)	211 (43.2 %)	72 (45.0 %)	97 (39.0 %)	34 (44.2 %)	308 (41.8 %)	106 (44.7 %)
Laboratory test results (median, SE)						
Serum PSA (ng/mL)	51.5 (23.2)	45.2 (54.7)	46.6 (23.2)	40.8 (22.3)	49.9 (17.2)	42.2 (37.7)
LDH (U/L)	190.0 (4.6)	194.0 (5.4)	187.5 (8.4)	192.0 (6.1)	190.0 (4.2)	193.5 (4.1)
Hemoglobin (g/dL)	12.9 (0.1)	12.6 (0.1)	12.7 (0.1)	12.7 (0.2)	12.9 (0.1)	12.7 (0.1)
Alkaline phosphatase (U/L)	103.0 (12.7)	101.0 (15.9)	104.0 (14.6)	93.0 (38.2)	103.0 (9.8)	100.0 (16.4)

^a^Included all subjects who had at least one of the three immune response assays measured on at least one visit

### Product parameters and analyses of antigen-specific T cells in the product

The sipuleucel-T final product (IMPACT only) comprised (median [25th, 75th percentile]): CD3^+^ T cells (62.3 % [51.8, 69.1 %]), APCs (18.3 % [12.3, 26.5 %], the majority of which were CD14^+^), CD56^+^ NK cells (11.0 % [6.9, 15.3 %]), and CD19^+^ B cells (4.7 % [2.6, 7.9 %]). The relative proportions of these cell types did not change across the treatment weeks.

#### Product parameters

**Fig. 1:**
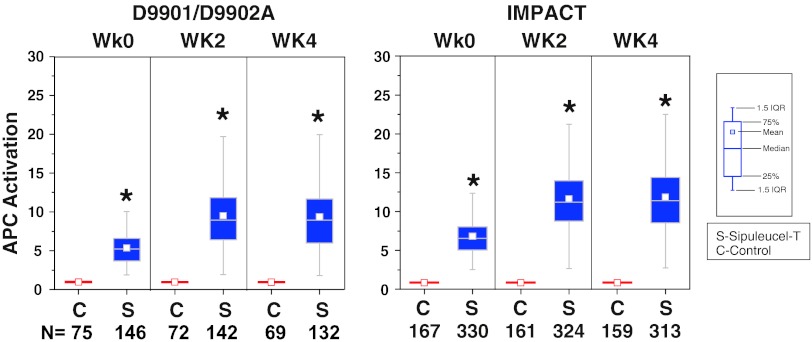
APC activation was characterized by CD54 upregulation on pre- and post-culture cells using flow cytometry. The increase in surface CD54 expression on large CD54^+^ cells post- versus pre-culture with PA2024 was expressed as an upregulation ratio of the average number of molecules on post-culture cells divided by the average number of molecules on pre-culture cells. *C* control, *S* sipuleucel-T. *P < 0.001 for sipuleucel-T versus control. Box plots represent 25th, 75th percentile range, whiskers represent 1.5× the interquartile range

In both the IMPACT and D9901/D9902A studies, APC activation was significantly greater with sipuleucel-T relative to control at weeks 0, 2, and 4 (Fig. 1). For sipuleucel-T, median [25th, 75th percentile] APC activation increased 6.2-fold [4.7, 7.7] in the first product, compared with 10.6-fold [7.8, 13.7] in the second and 10.5-fold [7.9, 13.7] in the third products (both *P* < 0.001 vs. first product, all studies pooled). The median cumulative APC activation with sipuleucel-T across the three dose preparations was 26.7 [21.5, 33.6]. TNC counts and number of APCs were consistent and not significantly different between doses prepared at weeks 0, 2, and 4; the median cumulative number [25th, 75th percentile] of TNCs and APCs across the three doses, pooled over the three studies were 9.70 × 10^9^ [6.97 × 10^9^, 13.55 × 10^9^], and 1.84 × 10^9^ [1.27 × 10^9^, 2.88 × 10^9^].

#### Antigen-specific T cells

**Fig. 2:**
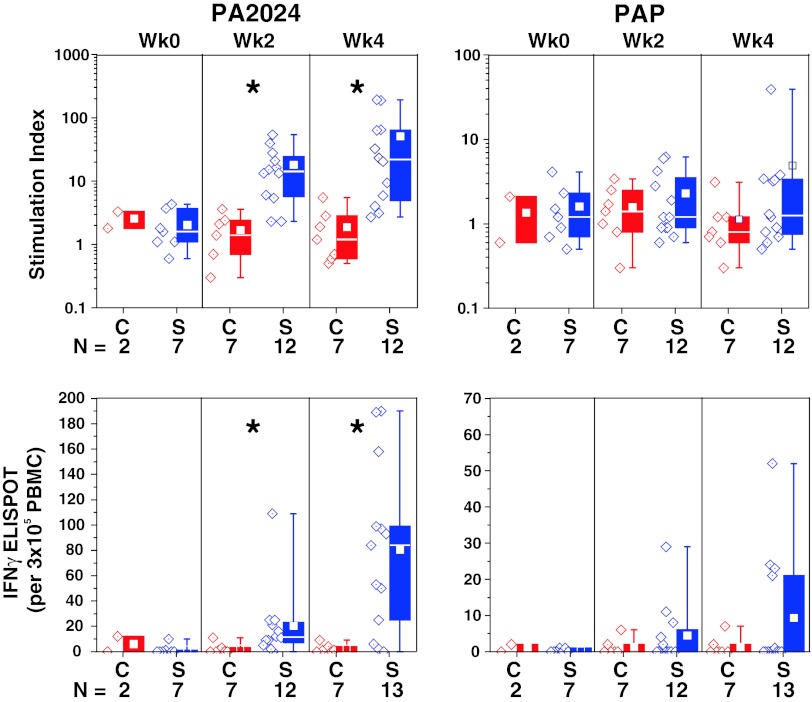
Immune responses were evaluated at treatment weeks 0, 2, and 4 for IMPACT subjects with remaining pre-culture cells. Antigen-specific T-cell proliferation was expressed as a stimulation index, the ratio of tritiated thymidine incorporation due to antigen stimuli compared to tritiated thymidine incorporation due to media alone. Antigen-specific T-cell memory was assessed by IFNγ ELISPOT, with each spot indicating a T cell that secretes IFNγ in response to stimuli, and the number of spots expressed as an integer of the number of PBMC plated/well of the ELISPOT plate (per 3 × 10^5^ PBMC). PBMC, peripheral blood mononuclear cells; PA2024, a fusion protein comprising PAP fused to granulocyte–macrophage colony-stimulating factor; PAP, prostatic acid phosphatase; *C* control; *S* sipuleucel-T. *P < 0.01 for sipuleucel-T versus control. Box plots for Stimulation Index represent 25th, 75th percentile range, whiskers represent 1st and 99th percentile; box plots for IFNγ ELISPOT represent SD, whiskers represent 1.5× SD

In the subset of IMPACT patients who were evaluated, significantly greater antigen-specific T cells, detected by proliferation and IFNγ ELISPOT responses against PA2024, were observed in dose preparations from the sipuleucel-T group relative to control at weeks 2 and 4, but not at week 0; the greatest values were observed at week 4 for both assays (Fig. 2). Some sipuleucel-T subjects also demonstrated responses to PAP that were greater than any of those observed in the control group and lower in magnitude than the anti-PA2024 responses.

#### Cytokine/chemokine production during manufacture

**Fig. 3:**
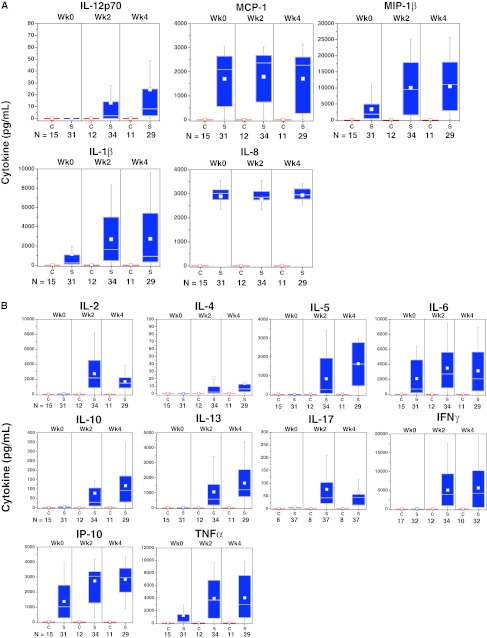

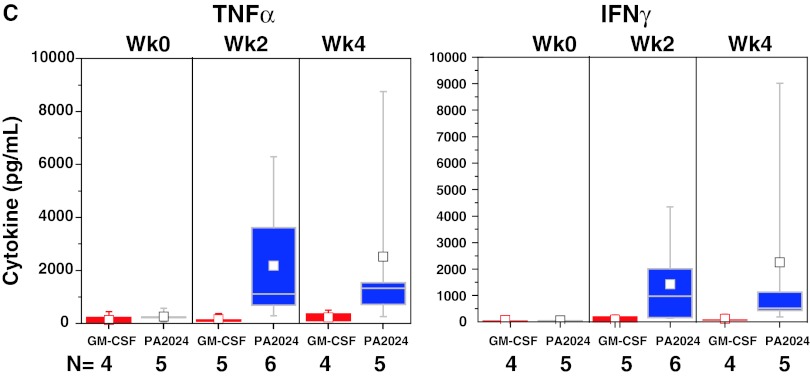
Chemokines and cytokines were measured in available post-culture supernatants of IMPACT subjects. Sipuleucel-T induced chemokines associated with APC activation (**a**) during manufacture of each dose, whereas cytokines associated with T-cell activation (**b**) were predominantly produced during manufacture of the second and third doses. **c** Restimulation of pre-culture cells, at each treatment week, with GM-CSF (Leukine) did not induce T-cell activation cytokines. *C* control; *S* sipuleucel-T. Box plots represent 25th, 75th percentile range, whiskers represent 1.5× the interquartile range

Cytokines produced by activated APCs were present in the culture media of sipuleucel-T, but not control, at weeks 0, 2, and 4 (Fig. 3a). Elevated levels of T-cell activation-associated cytokines were observed in the culture medium during the manufacture of the second and third doses (Fig. 3b). T-cell activation cytokines were not induced when pre-culture cells were cultured with GM-CSF alone (Fig. 3c).

### Characterization of peripheral immune responses

**Fig. 4:**
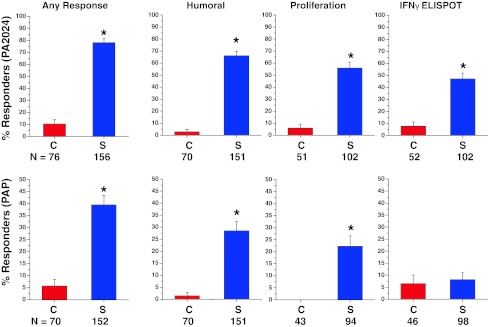
Immune responses were determined at baseline (week 0) as well as 6, 14, and 26 weeks following the first product infusion in a subset of patients enrolled in IMPACT. The percent (SE) of subjects with positive responses to antigen-specific antibody (ELISA), memory IFNγ ELISPOT, and T-cell proliferation are summarized. The positive threshold for antibody response was defined as a titer >400. The threshold for T-cell proliferation responders was defined as an SI > 12 for the PA2024 antigen and >8 for the PAP antigen. A positive IFNγ ELISPOT response was defined as >10 spots for the PA2024 antigen and >40 spots for the PAP antigen, per 3 × 10^5^ PBMC. *P < 0.001 for sipuleucel-T versus control

A positive humoral or cellular immune response to PA2024 and/or PAP in any post-baseline assay was observed in 78.8 % (123/156) of sipuleucel-T subjects compared with 13.2 % (10/76) of control subjects. An immune response to PA2024 was observed in 78.2 % (122/156) of sipuleucel-T subjects versus 10.5 % (8/76) of control subjects, and a response to PAP was observed in 39.5 % (60/152) of sipuleucel-T subjects versus 5.7 % (4/70) of control subjects (Fig. 4).

Sipuleucel-T treatment generated PA2024- and/or PAP-specific humoral responses in a majority of subjects (68 %; 102/151), compared with 3 % (2/70) of control subjects. Anti-PA2024 and anti-PAP antibody titers were greater in the sipuleucel-T group compared with control at all post-baseline time points (*P* < 0.001), with a positive response still evident 26 weeks after initial treatment in 53.2 % (PA2024) and 17.7 % (PAP) of subjects (Supplementary Figure 2A). The magnitude of PA2024- and PAP-specific IgM antibodies peaked at week 6 and remained detectable through week 26, while anti-PA2024 and anti-PAP IgG antibodies were detected at week 6 and further increased at weeks 14 and 26 (Supplementary Figure 2B).

Sipuleucel-T treatment also elicited PA2024- and/or PAP-specific cellular responses in a majority of subjects (60 % [61/102] T-cell proliferation; 48 % [49/102] IFNγ ELISPOT), compared with a positive response in control subjects of 6 % (3/51) for T-cell proliferation and 13 % [7/52] for IFNγ ELISPOT. PA2024-specific T-cell proliferation and IFNγ ELISPOT were significantly greater with sipuleucel-T at all post-baseline time points (*P* < 0.05; Supplementary Figure 3), and significantly more sipuleucel-T subjects responded to each assay (Fig. 4). The magnitude of PAP-specific T-cell proliferation was not significantly different between groups (Supplementary Figure 3); however, only sipuleucel-T subjects demonstrated a positive proliferation response to PAP (Fig. 4). PAP-specific IFNγ ELISPOT results and responder frequency were not significantly different between groups at any time point (Fig. 4; Supplementary Figure 3).

### Correlations of overall survival with product parameters and peripheral immune response

**Fig. 5:**
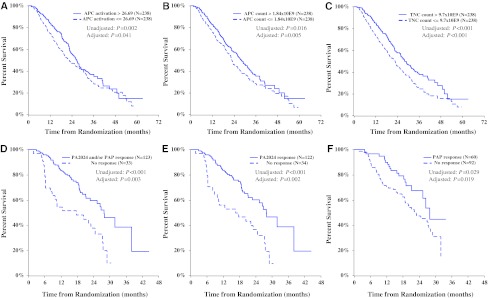
Overall survival assessed by product characteristics (**a**, **b**, **c**; Kaplan–Meier survival plots for sipuleucel-T subjects with product parameters above vs. below the median value; *P* values from analysis of each parameter as a continuous measure) and antigen-specific immune response (**d**, **e**, **f**; Kaplan–Meier survival plots for sipuleucel-T responders and non-responders for a subset of IMPACT subjects). **a** Cumulative APC activation value [HR = 0.76 (95 % CI: 0.58, 0.99)]. **b** Cumulative CD54^+^ cell count [HR = 0.79 (95 % CI: 0.68, 0.93)]. **c** Cumulative TNC count [HR = 0.71 (95 % CI: 0.59, 0.87)]. **d** Response to PA2024 or PAP in at least one of the three immune response assays [HR = 0.47 (95 % CI: 0.29, 0.78)]; **e** Response to PA2024 in at least one of the three immune response assays [HR = 0.46 (95 % CI: 0.28, 0.76)]; **f** Response to PAP in at least one of the three immune response assays [HR = 0.53 (95 % CI: 0.31, 0.90)]. HR < 1 indicates reduced risk of death with greater cumulative product parameter values (**a**, **b**, **c**). Hazard ratios and 95 % confidence intervals from analysis of the natural logarithm of each parameter as a continuous measure in a Cox regression model, stratified by study, and adjusted for baseline PSA and LDH. *P* values are from analyses with and without adjustment for baseline PSA and LDH. HR < 1 indicates reduced risk of death with presence of an immune response (**d**, **e**, **f**). Immune responder cohorts were defined as described in Methods. Hazard ratios and 95 % confidence intervals from a Cox regression model, adjusted for baseline PSA and LDH. *P* values are from analyses with and without adjustment for baseline PSA and LDH

In sipuleucel-T treated subjects, a positive correlation was observed between OS and cumulative APC activation, APC count, and TNC count (pooled studies; Fig. 5). These correlations remained after adjusting for baseline prognostic factors (PSA and LDH) that are independently correlated with OS [14]. For a graphical depiction of these correlations, Kaplan–Meier curves were generated for sipuleucel-T subjects with cumulative product parameters greater than versus less than or equal to the median value (Fig. 5a–c). OS was also significantly correlated with the development of at least one post-baseline peripheral immune response to PA2024 or PAP [HR = 0.47 (95 % CI: 0.29, 0.78) *P* = 0.003; Fig. 5d], to PA2024 [HR = 0.46 (95 % CI: 0.28, 0.76) *P* = 0.002; Fig. 5e], and to PAP [HR = 0.53 (95 % CI: 0.31, 0.90) *P* = 0.019; Fig. 5f]. The strongest correlation between OS and the development of a post-baseline immune response to PA2024 at any time point was observed with antibody responses [HR = 0.42 (95 % CI: 0.26, 0.67) *P* < 0.001], while the correlation with IFNγ ELISPOT at any time point approached statistical significance [HR = 0.55 (95 % CI: 0.28, 1.08) *P* = 0.08]; T-cell proliferation response did not significantly correlate with OS.

## Discussion

Sipuleucel-T, the first autologous cellular immunotherapy to be FDA-approved for the treatment of cancer, is manufactured from a patient’s own PBMCs obtained during leukapheresis. The mononuclear cells removed during leukapheresis constitute only a small percentage of the body’s total pool of lymphocytes [22–24] and are rapidly replenished [25] such that median cell counts were within normal ranges 2, 10, and 22 weeks after the third leukapheresis procedure [26, 27]. The PBMCs are cultured with the recombinant PAP-GM-CSF fusion antigen (PA2024), which is processed by APCs and presented as PAP epitopes to PAP-specific T cells [18]. The proportion of cell subtypes remains constant throughout the manufacturing process. The data presented here support the proposed mechanism of action: ex vivo-activated APCs from the first sipuleucel-T infusion engage the immune system in vivo in a manner similar to a classical vaccine-mediated memory response, where the first infusion primes the immune system and subsequent infusions boost the response.

Activation of APCs, as measured by CD54 upregulation, was evident in the first dose (week 0) in PA2024-cultured cells and further increased in the second (week 2) and third (week 4) doses. The supposition that the first infusion of activated, antigen-loaded APCs primes T cells in vivo is supported by evidence of antigen-specific T-cell proliferation and IFNγ ELISPOT activity in pre-culture cells obtained at weeks 2 and 4 (but not week 0), as well as the presence of T-cell activation-associated cytokines in the second and third doses of sipuleucel-T. While APCs do not have anamnestic properties, the presence of cytokines produced by activated T cells, such as TNFα, is known to further activate APCs and induce the expression of cytokines associated with APC activation (e.g., IL-1β) [28, 29]. Thus, the prime-boost pattern that was also detected for APC activation and associated cytokines could be due to signals from antigen-specific T cells re-stimulated with antigen during preparation of the second and third doses. Of note, the fact that re-stimulation of pre-culture cells with GM-CSF failed to induce cytokines associated with activated T cells supports the premise that GM-CSF alone is not responsible for the observed antigen-specific immune responses; this is consistent with preclinical findings [30].

TH1 cytokines (e.g., IFNγ, TNFα) in the product were present at high levels in comparison with IL-4, the classical marker of TH2 cells, but the presence of TH2 cytokines IL-5 and IL-13 implies that both TH1 and TH2 cells were activated in an antigen-specific manner. This is consistent with the observation that sipuleucel-T treatment induced both cellular and humoral responses. Intriguingly, IL-17 was also produced, suggesting the activation of TH17 cells, a TH subset known to have a pivotal role in mediating autoimmune responses [31, 32]. In addition, while IL-10 was also detectable, the relative amount of this T-cell-suppressive cytokine was markedly less than that of cytokines known to drive T-cell expansion, such as IL-2, IFNγ, and TNFα.

These data demonstrate that sipuleucel-T engages the immune system early in treatment and generates robust and persistent in vivo antigen-specific cellular and humoral immunity. In T-cell proliferative antigen recall assays, a pertinent measure of immunological responsiveness, PA2024-specific responses were present in the majority of sipuleucel-T-treated subjects with the magnitude of the response sustained through at least week 26. Furthermore, the IFNγ ELISPOT responses detected in the sipuleucel-T group at week 26 are indicative of persistent PA2024-specific memory T cells [33]. Finally, the development of early IgM responses followed by isotype switching to IgG antibody production is characteristic of memory plasma cell development and suggests the engagement of T cells and the establishment of long-lasting humoral immunity.

Immune responses to PAP were detectable but were less frequent and lower in magnitude than responses to the immunizing antigen (PA2024). Several factors may contribute to these findings. First, PA2024 was tailored to be more easily taken up and effectively processed by APCs and is larger (containing more epitopes) than PAP. Furthermore, the additional epitopes in PA2024 include the linker between GM-CSF and PAP, a neoepitope that may be more immunogenic than the epitopes to PAP. Finally, it should also be noted that tumor antigen-specific effector T cells may transit out of the peripheral blood, which is used as the source of PBMCs for the manufacture of sipuleucel-T and for immune monitoring, complicating comparisons between responses. Nevertheless, the fact that anti-PAP antibodies were detected in subjects treated with sipuleucel-T when no such antibodies could be identified in the pre-treatment specimens and that PAP-specific T-cell responders were observed only in the sipuleucel-T group supports the supposition that sipuleucel stimulates antigen-specific B- and T-cell responses that target prostate cancer.

The positive correlation observed between key product parameters, such as APC activation, and OS suggests that the broad engagement of the immune system by sipuleucel-T contributes to the survival benefit associated with therapy. Moreover, patients who developed a peripheral immune response to sipuleucel-T had improved OS. The strongest relationship between prolonged OS and long-term immune response was observed with PA2024-specific antibody and IFNγ ELISPOT responses. The absence of a significant correlation between OS and T-cell proliferation may be due to the non-specific nature of the T-cell proliferation assay (i.e., it does not distinguish between biologically functional vs. non-functional effector memory cells). As such, the IFNγ ELISPOT assay, which is a direct measure of the establishment of functional antigen-specific memory T cells, may be a better indicator of relevant immune response.

Limitations of this study include the availability of immune response data for only a subset of patients, and the post hoc correlative nature of the analyses. In addition, the correlations between immune parameters and increased OS could result from healthier subjects with an improved probability of survival being more likely to generate robust immune responses. However, the persistence of the correlations between these immune markers and OS following adjustment for baseline prognostic factors argues against this hypothesis, as does the fact that humoral recall response to tetanus did not change following treatment and was not different between the control and treatment groups (data on file; Dendreon). In addition, prior studies demonstrated no differences in baseline T-cell proliferation response to influenza between patients who did or did not subsequently develop an immune response to PAP [34]. Similarly, previous work in follicular lymphoma failed to demonstrate a correlation between anti-idiotype immune response in response to immunization and baseline prognostic factors or the ability to respond to KLH [35].

In summary, the data presented here demonstrate that ex vivo APC activation with sipuleucel-T induces a robust and long-lived in vivo immune response profile characteristic of immunological memory. Both cellular and humoral responses were evident, and the response status of subjects as well as the magnitude of the immune response correlated with OS. These data are consistent with sipuleucel-T’s intended mechanism of action: to generate antigen-specific immune responses that target prostate cancer. A recent study of sipuleucel-T administered prior to radical prostatectomy demonstrating increases in T cells at the tumor interface compared to the pre-treatment biopsy [36] provides further evidence in support of this mechanism of action. Given the increasing availability of novel immunomodulatory agents, combination studies with sipuleucel-T are warranted; augmentation of the immune parameters described herein would provide a strong rationale for larger studies to demonstrate increased clinical efficacy.

## Electronic supplementary material

Below is the link to the electronic supplementary material.

Supplementary Figure 1. Product characteristics were assessed as lot release parameters from sipuleucel-T manufacture at Wks 0, 2, and 4. Immune responses were assessed from available pre-culture cells during sipuleucel-T treatment at Wks 0, 2, and 4 and from blood samples at baseline (Wk 0), and at Wks 6, 14, and 26 weeks after the first infusion. Wk, week, ELISA, enzyme-linked immunosorbent assay, ELISPOT, enzyme-linked immunosorbent spot assay. (PPTX 57 kb)

Supplementary Figure 2. A. Antigen-specific antibody ELISA (IgM+IgG) in sipuleucel-T and control subjects before and after treatment. The amount of antigen-specific antibodies in serum was expressed as a reciprocal of the last dilution that yielded a signal equivalent to the assay control. Subjects with responses >400 were considered positive and the percentages of positive responders at each time point are listed below each time point on the x-axis (% Resp Freq). B. Antigen-specific antibody ELISA differentiating IgM and IgG from those subjects who gave positive anti-PA2024 and anti-PAPtiters, and had serum for assay. PA2024, a fusion protein comprising prostatic acid phosphatase (PAP) fused to granulocyte-macrophage colony-stimulating factor; PAP, prostatic acid phosphatase; C, control; S, sipuleucel-T. * = P<0.001 for sipuleucel-T vs. control. (PPTX 78 kb)

Supplementary Figure 3. Antigen-specific T-cell proliferation was expressed as a stimulation index (SI), the ratio of tritiated thymidine incorporation due to antigen stimuli compared to tritiated thymidine incorporation due to media alone. The positive threshold for T-cell proliferation was defined as an SI >12 for the PA2024 antigen and >8 for the PAP antigen. Antigen-specific T-cell memory was assessed by IFNγ ELISPOT, with each spot indicating a T cell that secretes IFNγ in response to stimuli, and the number of spots expressed as an integer of the number of PBMC plated/well of the ELISPOT plate. A positive IFNγ ELISPOT response was defined as >10 spots for the PA2024 antigen, and >40 spots for the PAP antigen, per 3x10^5^ PBMC. % Resp Freq, (Responder Frequency) percentage of patients categorized as a responder for each respective time point; PBMC, peripheral blood mononuclear cell; PA2024, a fusion protein comprising prostatic acid phosphatase (PAP) fused to granulocyte-macrophage colony-stimulating factor; PAP, prostatic acid phosphatase; C, control; S, sipuleucel-T. * = P<0.001 for sipuleucel-T vs. control; # = P<0.05 for sipuleucel-T vs. control. (PPTX 74 kb)
