# Supplementary figures and images for: Sipuleucel-T immune parameters correlate with survival: an analysis of the randomized phase 3 clinical trials in men with castration-resistant prostate cancer
Source: Cancer Immunol Immunother. 2012 Aug 3;62(1):137–47. doi: 10.1007/s00262-012-1317-2 (PMC3541926; doi:10.1007/s00262-012-1317-2)

## Slide 1
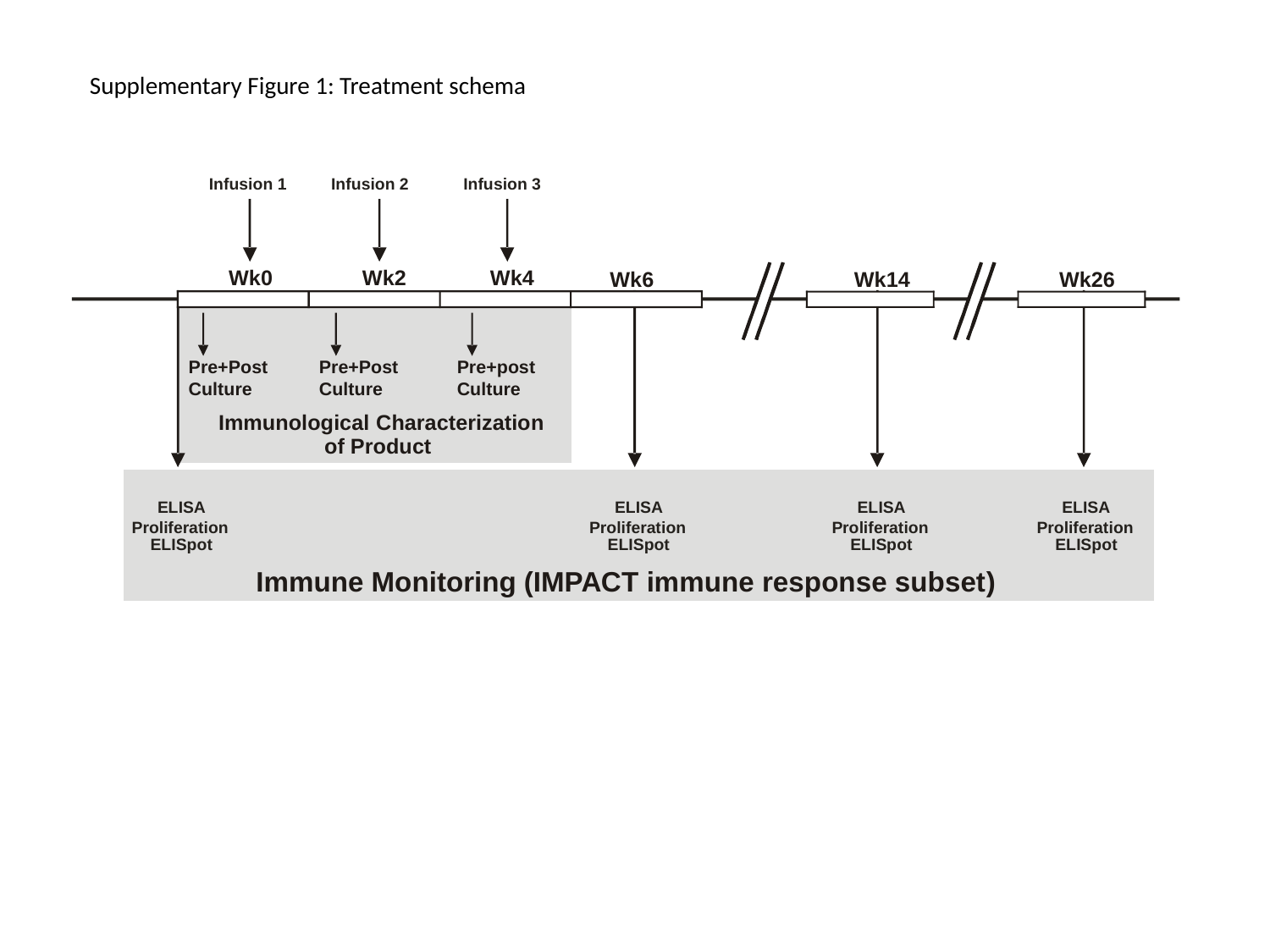

Supplementary Figure 1: Treatment schema

Supplement: Supplementary file 1 — Supplementary Figure 1. Product characteristics were assessed as lot release parameters from sipuleucel-T manufacture at Wks 0, 2, and 4. Immune responses were assessed from available pre-culture cells during sipuleucel-T treatment at Wks 0, 2, and 4 and from blood samples at baseline (Wk 0), and at Wks 6, 14, and 26 weeks after the first infusion. Wk, week, ELISA, enzyme-linked immunosorbent assay, ELISPOT, enzyme-linked immunosorbent spot assay. (PPTX 57 kb) [file 262_2012_1317_MOESM1_ESM.pptx]

## Slide 1
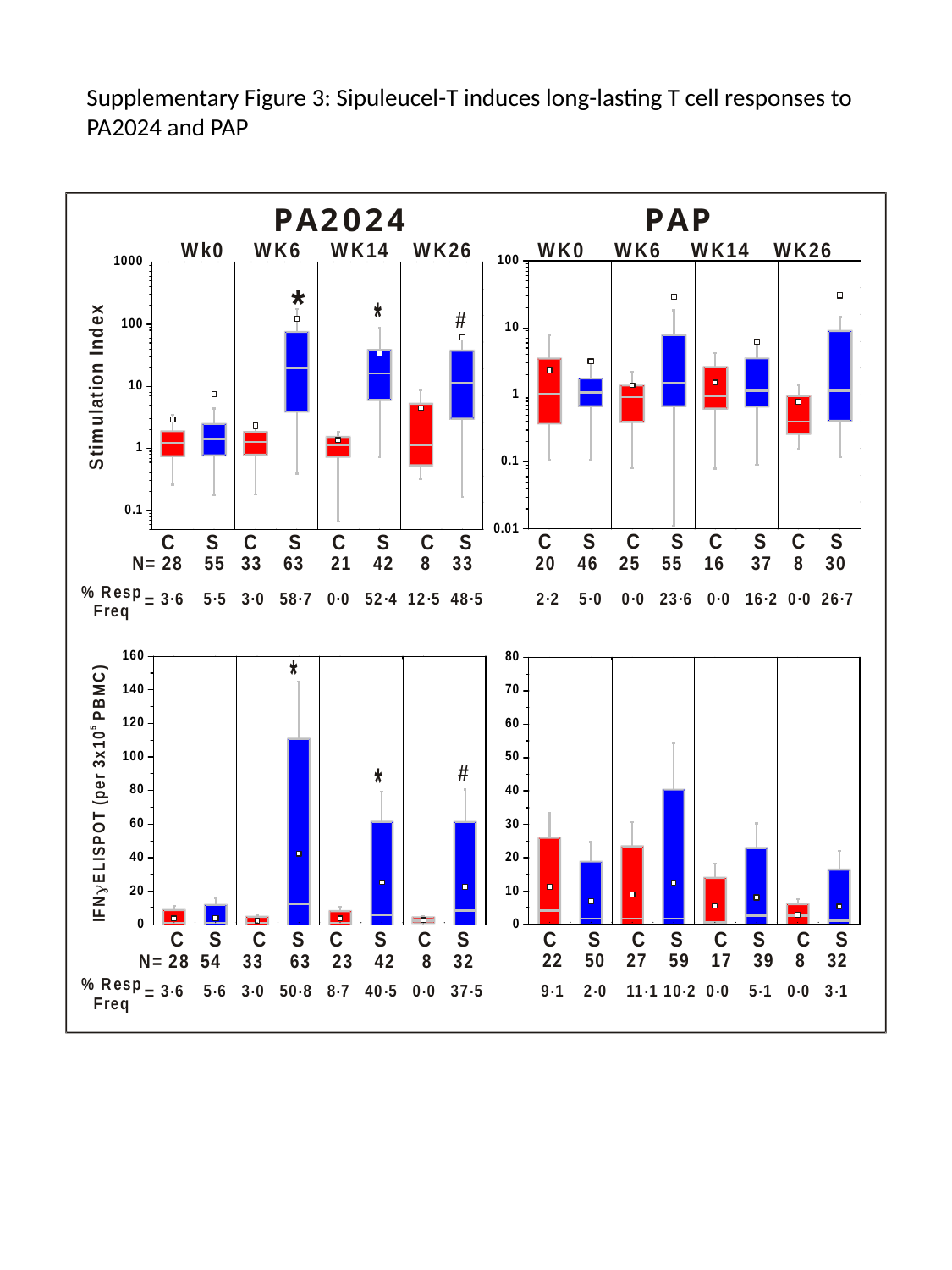

Supplementary Figure 3: Sipuleucel-T induces long-lasting T cell responses to PA2024 and PAP

Supplement: Supplementary file 3 — Supplementary Figure 3. Antigen-specific T-cell proliferation was expressed as a stimulation index (SI), the ratio of tritiated thymidine incorporation due to antigen stimuli compared to tritiated thymidine incorporation due to media alone. The positive threshold for T-cell proliferation was defined as an SI >12 for the PA2024 antigen and >8 for the PAP antigen. Antigen-specific T-cell memory was assessed by IFNγ ELISPOT, with each spot indicating a T cell that secretes IFNγ in response to stimuli, and the number of spots expressed as an integer of the number of PBMC plated/well of the ELISPOT plate. A positive IFNγ ELISPOT response was defined as >10 spots for the PA2024 antigen, and >40 spots for the PAP antigen, per 3x10^5^ PBMC. % Resp Freq, (Responder Frequency) percentage of patients categorized as a responder for each respective time point; PBMC, peripheral blood mononuclear cell; PA2024, a fusion protein comprising prostatic acid phosphatase (PAP) fused to granulocyte-macrophage colony-stimulating factor; PAP, prostatic acid phosphatase; C, control; S, sipuleucel-T. * = P<0.001 for sipuleucel-T vs. control; # = P<0.05 for sipuleucel-T vs. control. (PPTX 74 kb) [file 262_2012_1317_MOESM3_ESM.pptx]
