# Supplementary material for: Sipuleucel-T immune parameters correlate with survival: an analysis of the randomized phase 3 clinical trials in men with castration-resistant prostate cancer
Source: Cancer Immunol Immunother. 2012 Aug 3;62(1):137–47. doi: 10.1007/s00262-012-1317-2 (PMC3541926; doi:10.1007/s00262-012-1317-2)
Supplement: Supplementary file 2 — Supplementary Figure 2. A. Antigen-specific antibody ELISA (IgM+IgG) in sipuleucel-T and control subjects before and after treatment. The amount of antigen-specific antibodies in serum was expressed as a reciprocal of the last dilution that yielded a signal equivalent to the assay control. Subjects with responses >400 were considered positive and the percentages of positive responders at each time point are listed below each time point on the x-axis (% Resp Freq). B. Antigen-specific antibody ELISA differentiating IgM and IgG from those subjects who gave positive anti-PA2024 and anti-PAPtiters, and had serum for assay. PA2024, a fusion protein comprising prostatic acid phosphatase (PAP) fused to granulocyte-macrophage colony-stimulating factor; PAP, prostatic acid phosphatase; C, control; S, sipuleucel-T. * = P<0.001 for sipuleucel-T vs. control. (PPTX 78 kb) [file 262_2012_1317_MOESM2_ESM.pptx]

## Slide 1
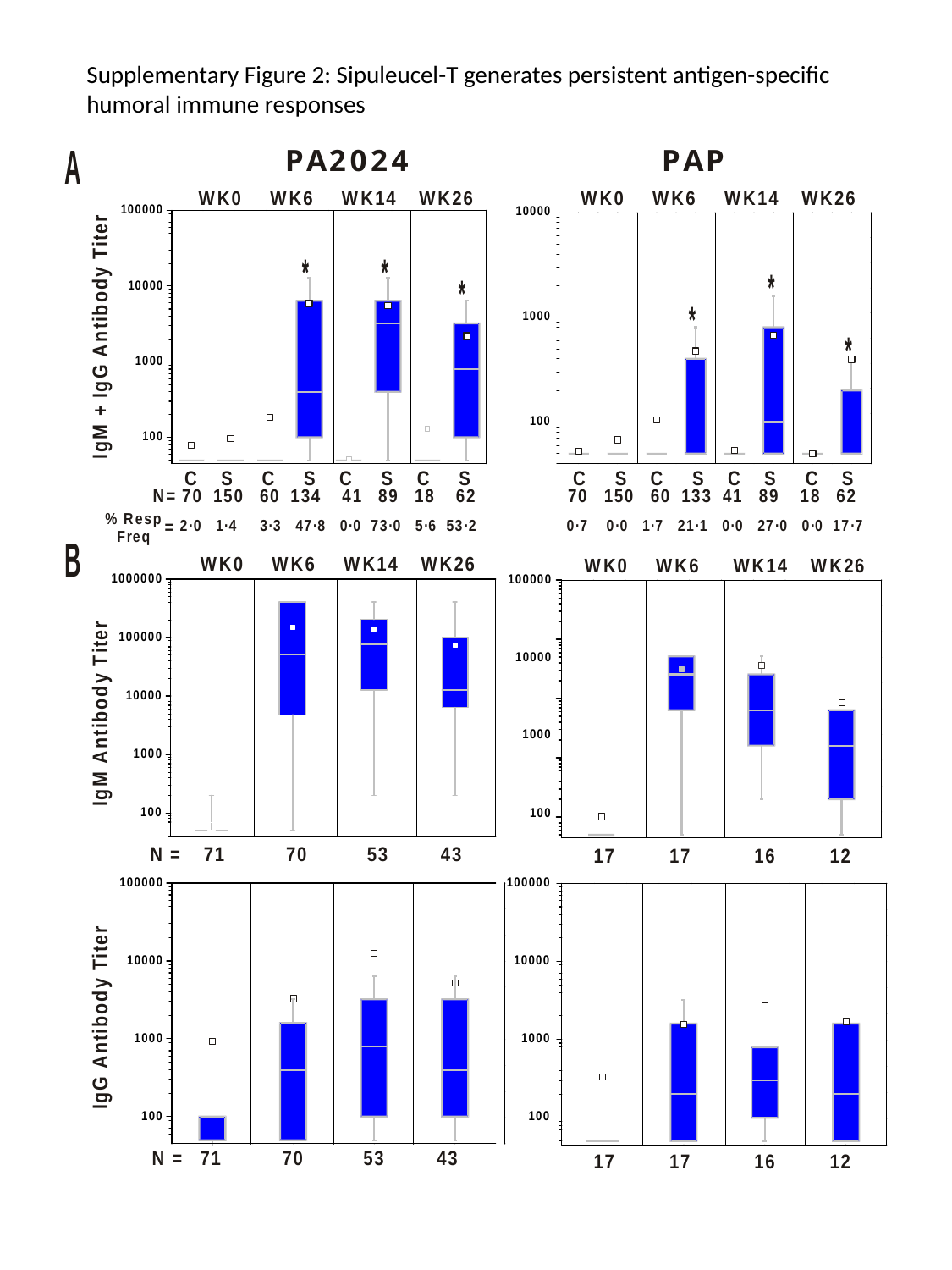

Supplementary Figure 2: Sipuleucel-T generates persistent antigen-specific humoral immune responses
